# *‘Pseudomonas saudiphocaensis’* sp. nov., a new bacterial species isolated from currency notes collected during the Hajj pilgrimage in 2012 at Makkah, Saudi Arabia

**DOI:** 10.1016/j.nmni.2016.12.004

**Published:** 2016-12-09

**Authors:** E.I. Azhar, A. Papadioti, F. Bibi, A.M. Ashshi, D. Raoult, E. Angelakis

**Affiliations:** 1) Special Infectious Agents Unit, King Fahd Medical Research Center, Jeddah, Saudi Arabia; 2) Department of Medical Laboratory Technology, Faculty of Applied Medical Sciences, King Abdulaziz University, Jeddah, Saudi Arabia; 3) Unité de Recherche sur les Maladies Infectieuses et Tropicales Emergentes, URMITE CNRS-IRD 198 UMR 6236, Aix Marseille Université, Faculté de Médecine, Marseille, France; 4) Department of Laboratory Medicine, Faculty of Applied Medical Science, Umm Al-Qura University, Makkah, Saudi Arabia

**Keywords:** Bank notes, culturomics, Hajj, *Pseudomonas saudiphocaensis*, Saudi Arabia

## Abstract

We report here the main characteristics of *‘Pseudomonas saudiphocaensis’* strain 20_BN^T^ (CSUR P1224), a new species of the *Pseudomonas* genus that was isolated from currency notes collected during the Hajj pilgrimage in 2012 at Makkah, Saudi Arabia.

Paper currency is commonly and routinely passed among individuals, and microbes can be spread on the surface of paper currency [1]. As a part of a wider culturomics study in Saudi Arabia, we isolated a new bacterium, strain 20_BN^T^, from currency notes collected during the Hajj pilgrimage in 2012 at Makkah, Saudi Arabia. Strain 20_BN^T^ was cultured in 5% sheep’s blood–enriched Columbia agar (bioMérieux, Marcy l’Etoile, France) for 2 days in an aerobic atmosphere at 37°C. On Columbia agar, colonies were yellow-transparent and round, with an average diameter of 1 mm. Growth was observed in aerobic and anaerobic conditions. The strain 20_BN^T^ is a Gram-negative, rod-shaped, motile catalase and oxidase-positive bacterium. Growth was observed in the range of 0 to 2% NaCl, with the optimum being 0.5% NaCl. Cells from fresh colonies grown on agar were examined by electron microscopy. A mean diameter of 0.5 μm and a mean length of 2.2 μm were estimated, as well as a single polar flagellum per cell. No identification was obtained for the strain 20_BN^T^ using our matrix-assisted laser desorption/ionization time-of-flight mass spectrometry (MALDI-TOF MS) screening on a MicroFlex spectrometer (Bruker Daltonics, Bremen, Germany) [2].

The complete 16S rRNA gene was sequenced using fD1-rP2 primers as previously described and using a 3130-XL sequencer (Applied Biosciences, Saint Aubin, France) [3]. Strain 20_BN^T^ exhibited a 98.3% sequence similarity with *Pseudomonas stutzeri* (NR103934.1), the phylogenetically closest species with standing in nomenclature (Fig. 1). Consequently it putatively classifies the strain 20_BN^T^ as a new member of the genus *Pseudomonas* within the family *Pseudomonadaceae* in the phylum *Proteobacteria.* The genus *Pseudomonas* was first created in 1894 by Migula, and an emended description of the genus *Pseudomonas* was proposed by Yang *et al.* in 2013 [4]. To date more than 200 species have been described (http://www.bacterio.cict.fr/c/pseudomonas.html). Members of the genus *Pseudomonas* are mostly environmental bacteria widely distributed in soil, water and air [5].

Strain 20_BN^T^ exhibited a 16S rRNA gene sequence divergence >1.3% with *P. stutzeri,* the closest related species with standing in nomenclature, which classifies it as a new representative of the *Pseudomonas* genus isolated from currency notes collected during the Hajj pilgrimage in 2012 at Makkah. As a result, we propose the creation of *‘Pseudomonas saudiphocaensis’* sp. nov., and strain 20_BN as the type strain.

## MALDI-TOF MS spectrum

The MALDI-TOF MS spectrum of 20_BN^T^ is available online (http://www.mediterranee-infection.com/article.php?laref=256&titre=urms-database).

## Nucleotide sequence accession number

The 16S rRNA gene sequence of the strain 20_BN^T^ was deposited in GenBank under accession number LK021121.

## Deposit in a culture collection

Strain 20_BN^T^ was deposited in the Collection de Souches de l’Unité des Rickettsies (CSUR, WDCM 875) under number P1224.

## Figures and Tables

**FIG. 1:**
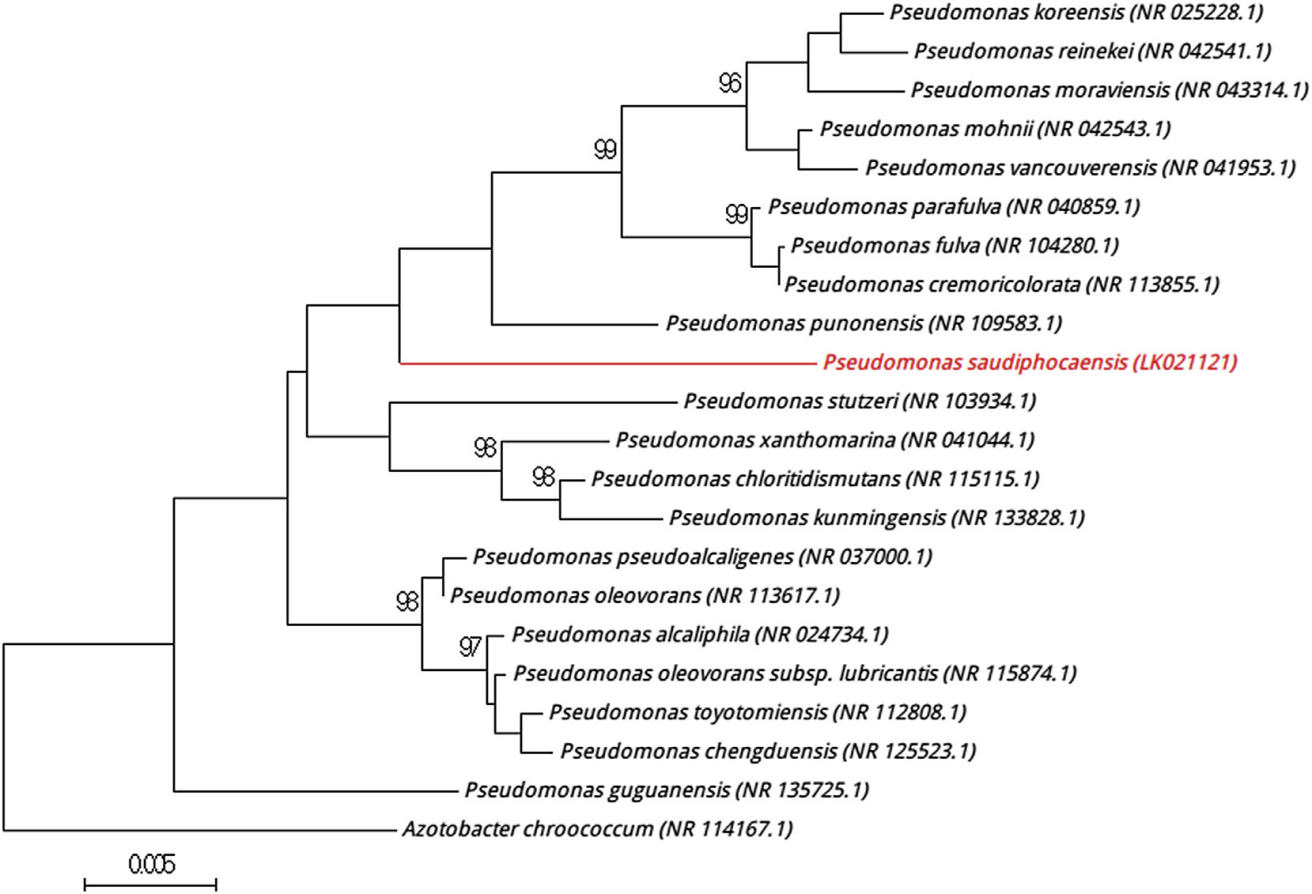
Phylogenetic tree highlighting position of *‘Pseudomonas saudiphocaensis’* relative to other phylogenetically closest members of *Pseudomonas* genus. Numbers at nodes are percentages of bootstrap values obtained by repeating analysis 500 times to generate majority consensus tree. Only values >95% are displayed. Scale bar represents 0.5% nucleotide sequence divergence.
